# Supplementary material for: The discovery of novel noncoding RNAs in 50 bacterial genomes
Source: Nucleic Acids Res. 2024 Apr 22;52(9):5152–65. doi: 10.1093/nar/gkae248 (PMC11109978; doi:10.1093/nar/gkae248)
Supplement: gkae248_Supplemental_Files [file gkae248_supplemental_files.zip › Narunsky 50 Genomes RNA Motifs Files.zip!/Publication/54-P_aestuarii/Figures/LRC-54-49-NC_011059_1_IGR_125_785140_785441.sto.pdf]

LRC-54-49-NC_011059_1_IGR_125_785140_785441.sto.r2r

5′-GUGAUGUUGGGGACAACGAGGCUGUGGUUGAUCUUUUUUCUCUAUAACCUAUAAAAAAGUGGUUUAAGCUCAAAGCGAAAAAAGCGUAAUUCUGAAAUAUAAGCUAAGCAAAGGAUUU●GAUGGCGGAGCGCAAAAGUGUUGGUAGAAUCAAGAGCAAUCAAGAGGGUACGGGGGCGUCUUAGCGCUGAGGUUUUCUGAUGGCUGUAUCGACCAAAAGAUUGUCGGCAAGGAGAAGAGAUGCGCUCUGCGUUGAGCUCAACAGGGGYUURU●●●AYAARCCCCUGUUGAGCUCAGGGAUG
